# Raman spectroscopic signatures of prostate cancer progression: correlation with gleason score

**DOI:** 10.1007/s11010-026-05647-2

**Published:** 2026-07-20

**Authors:** Samaneh Ghazanfarpour, Rahul Kumar Das, Satish Sharma, Stanley A. Schwartz, Kent Chevli, Wilfrido Mojica, Anna Sharikova, Supriya D. Mahajan, Alexander Khmaladze, Ravikumar Aalinkeel

**Affiliations:** 1 Department of Physics, University at Albany SUNY, 1400 Washington Avenue, Albany, NY 12222 USA; 2 Department of Medicine, Jacobs School of Medicine and Biomedical Sciences, Clinical Translational Research Center, University at Buffalo, 875 Ellicott Street, Buffalo, NY 14203 USA; 3 Department of Urology, Jacobs School of Medicine and Biomedical Sciences, University at Buffalo, Buffalo, NY 14203 USA; 4 Department of Pathology & Anatomical Sciences, Jacobs School of Medicine and Biomedical Sciences, University at Buffalo, 955 Main Street, Buffalo, NY 14203 USA; 5 Department of Medicine, Division of Allergy, Immunology, and Rheumatology Jacobs School of Medicine and Biomedical Sciences, University at Buffalo, Buffalo, NY USA

**Keywords:** Gleason score, Prostate Cancer (CaP), Prostate specific membrane antigen (PSMA), Formalin-fixed paraffin-embedded (FFPE), Metastasis, Tumor microenvironment (TME), Raman spectroscopy (RS), Singular Value Decomposition (SVD)

## Abstract

Prostate cancer (CaP) is a heterogeneous malignancy, and its grading via Gleason scoring is essential for prognosis and therapeutic decision‑making. However, traditional histopathological methods are limited in detecting subtle biochemical variations. This study applies Raman spectroscopy (RS) to formalin‑fixed paraffin‑embedded (FFPE) CaP tissues of varying Gleason scores to uncover biochemical signatures associated with tumor aggressiveness. Raman spectral analysis reveals distinct molecular alterations particularly in lipid, protein, and nucleic acid content that differ across Gleason patterns reflecting grade‑associated biochemical differences rather than any biological progression between patterns. Singular Value Decomposition (SVD) of Raman data enables discrimination between benign prostate tissue and higher‑grade cancer (Gleason ≥ 7) included in this study and stratifies the analyzed Gleason patterns. Our goal in this initial study was not to define definitive Raman biomarkers but to evaluate whether consistent biochemical differences across Gleason grades could be detected in FFPE tissue. Our findings support the potential of Raman spectroscopy as a label‑free, non‑destructive adjunct to conventional histopathology for improved CaP stratification.

## Highlights

- Raman spectroscopy (RS) revealed progressive biochemical alterations in CaP tissues across Gleason scores 7–10, reflecting metabolic reprogramming, structural remodeling, and genomic activation.
- Lipid-associated bands (987, 1090, 1437, 1462 cm⁻¹) showed reduced intensity in high-grade tumors, indicating disrupted lipid metabolism and membrane remodeling.
- Carbohydrate-related bands (1106, 1122, 1140 cm⁻¹) progressively increased with Gleason score, consistent with elevated glucose uptake and glycosylation activity.
- Protein-associated amide bands (1205, 1237, 1637–1687 cm⁻¹) exhibited spectral shifts, highlighting enhanced protein synthesis, denaturation, and secondary structure changes in aggressive CaP.
- Nucleic acid-associated bands (1044, 1337, 1584 cm⁻¹) intensified with tumor grade, reflecting elevated genomic activity and DNA replication.
- RS combined with multivariate statistical analysis achieved accurate classification of CaP grades, supporting its integration into histopathological workflows for non-invasive, label-free tumor grading.

## Introduction

Prostate cancer (CaP) is the second most common malignancy in men worldwide and displays a broad spectrum of clinical behaviors that complicate disease management [1–4]. The Gleason score, derived from glandular architectural patterns, remains the cornerstone for grading prostate adenocarcinoma and is a strong predictor of tumor aggressiveness, metastatic potential, and cancer‑specific survival [5–7]. Serum prostate‑specific antigen (PSA) levels complement Gleason grading, yet the strength and consistency of this association across discrete Gleason grade groups remain incompletely defined [8–10]. Despite its clinical utility, Gleason grading does not fully capture the biochemical heterogeneity of CaP [11], and its subjective nature contributes to inter‑observer variability as high as 40%, particularly when distinguishing intermediate‑grade (Gleason 7) from high‑grade (Gleason 8–10) disease [10, 11].

Gleason score 7 encompasses two biologically distinct subgroups: 3 + 4=7 and 4 + 3=7; with markedly different prognostic implications. Tumors with a predominant pattern 3 (3 + 4=7) generally exhibit more favorable outcomes, whereas those with a predominant pattern 4 (4 + 3=7) behave more aggressively and often resemble high‑grade disease [12–15]. Gleason scores 8–10 reflect poorly differentiated tumor architecture and are consistently associated with increased risks of progression, metastasis, and mortality [16]. This heterogeneity complicates risk stratification and treatment planning, often necessitating adjunctive tools such as genomic classifiers and multiparametric MRI [15, 16]. Accurate grading is therefore essential to avoid under‑ or over‑treatment and to guide appropriate therapeutic decisions [17, 18].

Optical spectroscopic technologies, particularly Raman spectroscopy (RS), have emerged as promising tools for refining CaP risk stratification by probing biochemical alterations that are not visible on routine histopathology [19–21]. RS is a label‑free vibrational technique capable of detecting subtle molecular changes in lipids, proteins, nucleic acids, and other biomolecules, generating a unique spectral fingerprint for each tissue type [20–22]. Multivariate analytical approaches such as singular value decomposition (SVD) and linear discriminant analysis (LDA) are commonly applied to Raman datasets to extract diagnostically relevant spectral features from complex biological samples [23–25].

A substantial body of work has explored RS for CaP detection and grading in vivo and ex vivo on fresh tissue. Probe‑based in vivo RS has demonstrated real‑time discrimination between benign and malignant prostate tissue and, in some studies, differentiation between low‑ and high‑risk disease during biopsy or surgical procedures [26–28]. Complementary ex vivo studies on freshly excised prostate specimens have shown that RS can distinguish cancerous from benign regions and identify biochemical signatures associated with increasing Gleason grade, including shifts in lipid saturation, protein secondary structure, and nucleic acid content. Parallel investigations on frozen and fixed prostate tissues have further advanced the field. Recent studies have demonstrated that Raman spectroscopy can reliably differentiate malignant from benign prostate tissue and detect clinically relevant biochemical differences, including with modern portable RS systems such as the one reported by van Breugel et al. (2023) [29, 30]. Subsequent work on frozen, formalin‑fixed, and paraffin‑embedded tissues has identified Raman biomarkers linked to tumor aggressiveness, extracapsular extension, and adverse pathological features [31–36]. These studies collectively highlight the diagnostic potential of RS across multiple tissue preparations, yet systematic evaluation of Raman biomarkers across well‑annotated archival FFPE cohorts spanning the full Gleason spectrum remains limited.

We recently demonstrated that pharmacologic inhibition of galectin‑3 (Gal‑3) induces significant Raman‑detectable alterations in lipids, proteins, and nucleic acids in CaP tissue, highlighting the sensitivity of RS to biochemical changes associated with tumor progression and therapeutic response [37]. Building on this foundation, the present study focuses on archival FFPE CaP tissue to address two key gaps:

(i) the identification of Raman spectral markers associated with discrete Gleason grade groups; including the clinically nuanced distinction between Gleason 3 + 4 and 4 + 3 and.
(ii) the evaluation of RS‑based multivariate models for differentiating intermediate‑ from high‑grade CaP in a retrospective, pathology‑linked cohort. Because FFPE blocks represent the most widely available clinical biospecimens, RS applied to FFPE tissue offers a powerful platform for retrospective biomarker discovery and for developing objective, reproducible tools to complement Gleason grading.

Our findings demonstrate that RS can differentiate CaP tissues across a range of Gleason scores and identify spectral markers indicative of tumor aggressiveness, supporting its potential role in enhancing the precision of CaP grading.

### Novelty of this study

Despite extensive Raman studies on prostate cancer including in vivo probe‑based measurements, fresh ex vivo analyses, and investigations on frozen or fixed tissues, few studies have systematically evaluated Raman biomarkers across a broad range of Gleason grades using archival FFPE specimens. Prior work has typically focused on binary discrimination (benign vs. malignant) or limited grade comparisons, whereas the clinically critical distinction between Gleason 7 (3 + 4 or 4 + 3), and high‑grade Gleason 8–10 disease remains underexplored. Our study addresses this gap by applying full‑spectrum RS with multivariate modeling to a well‑annotated FFPE cohort spanning the full Gleason spectrum, enabling direct comparison of Raman signatures across clinically relevant grade groups and identifying spectral markers associated with tumor aggressiveness. This positions our work as a novel contribution that complements and extends existing Raman literature in CaP.

## Materials and methods

### Sample collection and preparation

CaP biopsy samples were obtained from the archived tissue bank of the Department of Urology at Western New York Urology Associates, Cheektowaga, NY-14,225. Patient tissue samples obtained after informed consent was obtained from patients, as per protocols and approval by our Institutional Review Borad (IRB). Tissue sampling was performed using spring-loaded or coaxial needles, typically 18-gauge, under transrectal ultrasound (TRUS) guidance. In some cases, 16-gauge needles were employed to yield larger cores, though cancer detection rates have been reported as comparable. The 18G needles produced cores approximately 0.8 mm in diameter and 15 mm in length. Section (4 μm thick) were mounted on calcium fluoride (CaF₂) slides and stored at − 80 °C until analysis. To preserve native molecular profiles, no staining, labeling, or chemical treatment was applied. None of the patients received preoperative therapy. All protocols were approved by the Ethics Committee of the Jacobs School of Medicine and Biomedical Sciences, Buffalo, NY, USA. The biopsies represented the following range of Gleason scores: Gleason 7 (3 + 4/4 + 3) **(G7)** – intermediate grade; Gleason 8–10 **(G8; G9; G10)** high grade.

For this study, a total of 20 independent biopsy cores were analyzed, obtained from 20 different patients. The cohort included:

• Gleason 7 (3 + 4 or 4 + 3): *n* = 4 cores.
• Gleason 8: *n* = 4 cores.
• Gleason 9: *n* = 4 cores.
• Gleason 10: *n* = 4 cores.
• Non‑cancerous controls: *n* = 4 cores.

Each biopsy core was formalin‑fixed, paraffin‑embedded (FFPE), and sectioned at 4 μm thickness. For Raman analysis, one section per core was mounted on calcium fluoride (CaF₂) slides and stored at − 80 °C. While formalin fixation and paraffin embedding inherently modify tissue biochemistry and contribute their own Raman signatures, we want to iterate that no additional staining or exogenous labelling was applied during Raman measurements.

**Fig. 1:**
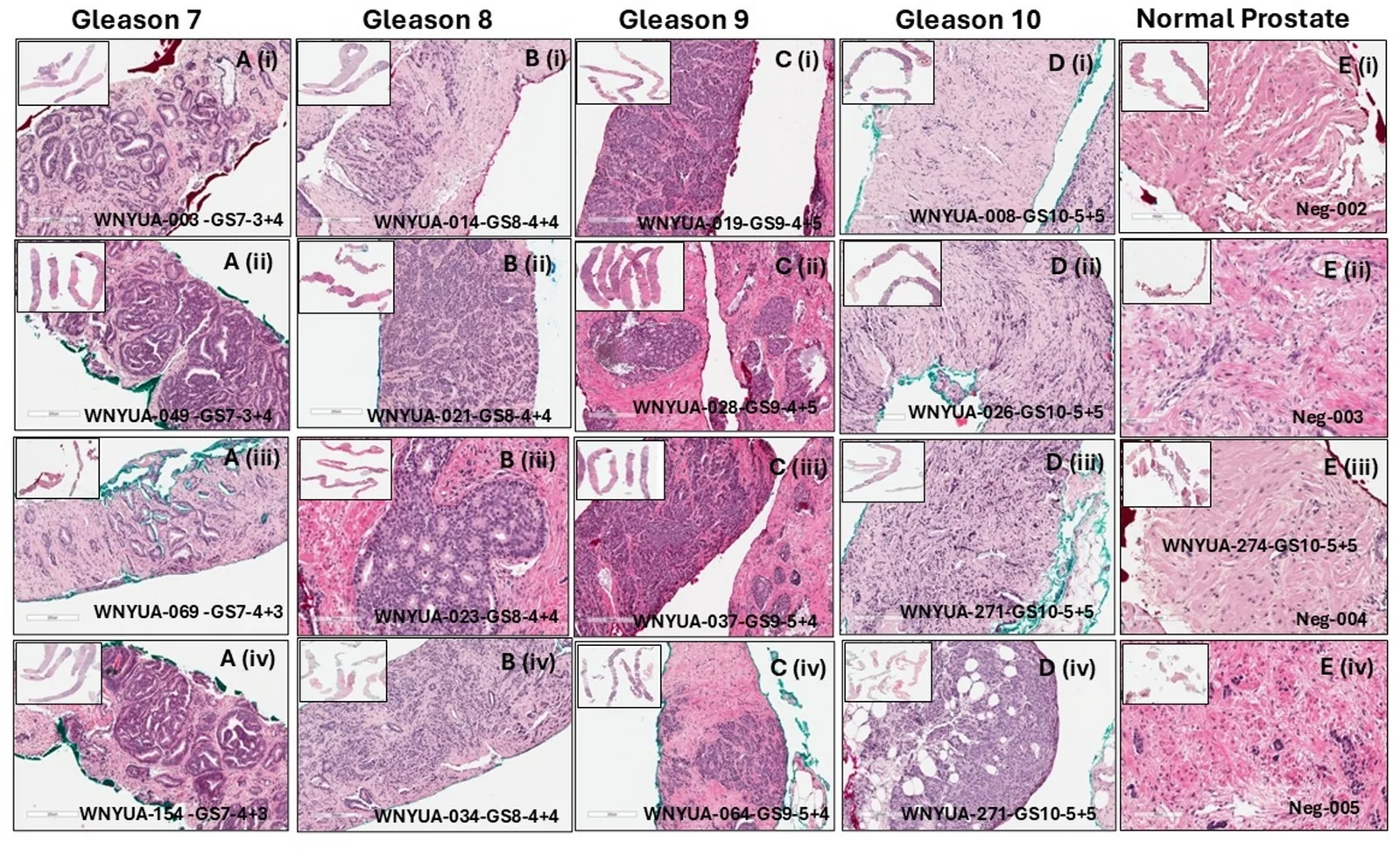
Representative H&E-stained sections of prostate tissue from all patient samples analyzed in the study (*n* = 4) in each category (**A** i-iv), Gleason score 7; CaP tissue with mixed well-formed and poorly formed glands. (**B** i-iv) Gleason score 8; CaP tissue displaying fused and cribriform glands with nuclear atypia. (**C** i-iv) Gleason score 9 CaP tissue showing solid sheets of tumor cells and loss of glandular structure. (**D** i-iv) Gleason score 10 CaP tissue with undifferentiated tumor cells in single-cell infiltrates and extensive necrosis. (**E** i-iv) Normal prostate tissue showing well-organized glandular architecture and uniform epithelial cells. Scale bars: 100 μm for each section, and Inlet image scale = 3 μm

Representative hematoxylin and eosin (H&E) stained sections of CaP tissues used in this study (Fig. 1), which show CaP tissue grades by a pathologist based on Gleason scores ranging from 7 to 10, demonstrate a progressive loss of glandular architecture and increasing cellular atypia compared to normal prostate tissue controls. Normal prostate glands exhibit well-organized acini lined by a uniform layer of epithelial cells with basally located nuclei and intact fibromuscular stroma. In contrast, Gleason score 7 tissues show a mixture of well-formed and poorly formed glands, while Gleason 8 sections reveal predominantly fused or cribriform glands with marked nuclear enlargement. Gleason scores 9 and 10 are characterized by a complete absence of glandular structures, with tumor cells arranged in solid sheets or single-cell infiltrates, accompanied by prominent nucleoli and areas of necrosis. These histological changes reflect increasing tumor aggressiveness and loss of differentiation with higher Gleason grades.

### Raman spectra acquisition of CaP tissue

Raman spectra were collected from each of the 20 biopsy cores. For every core, 40 spectra were acquired from spatially distinct regions of the same 4‑µm FFPE section to capture intratumoral biochemical heterogeneity while avoiding repeated sampling of the same microscopic area.

Thus, the total number of spectra per group was:

• Gleason 7: 4 cores × 40 spectra = 160 spectra.
• Gleason 8: 4 cores × 40 spectra = 160 spectra.
• Gleason 9: 4 cores × 40 spectra = 160 spectra.
• Gleason 10: 4 cores × 40 spectra = 160 spectra.
• Controls: 4 cores × 40 spectra = 160 spectra.

Total spectra used for LDA analysis: 800. Only spectra from pathologist‑verified cancerous regions (or benign regions for controls) were included.

Raman spectra were acquired by a commercial Raman micro-spectroscope (HORIBA XploRA PLUS) equipped with a 1024 × 256 TE air-cooled CCD chip (pixel size 26 μm, temperature − 60 °C). Spectra were acquired using a 532 nm laser operating at a power of 0.065 W, with an 1800 grooves/mm grating, a slit width of 100 μm, and a pinhole diameter of 100 μm. Each spectrum was recorded with an acquisition time of 30 s and three accumulations to enhance signal quality. A 40x objective was employed for focusing, and a total of 40 spectra per sample were collected for analysis. Spectra were acquired over 950–1800 cm⁻¹ (fingerprint region).

### Assignment of gleason grade to Raman spectra

Each Raman spectrum was acquired from a spatially localized region within a biopsy core. To ensure accurate histopathological correspondence, a board‑certified pathologist reviewed the H&E‑stained adjacent sections and identified regions representing the dominant Gleason patterns that contributed to the final Gleason score for each core. Raman acquisition points were selected exclusively within these pathologist‑annotated regions. Thus, although each Raman measurement reflects a small local area, the spectra were assigned the Gleason score of the corresponding biopsy core because the sampled regions were confirmed to represent the predominant Gleason pattern(s) defining that score. This approach ensured that all spectra used for analysis were derived from histologically validated cancerous regions consistent with the assigned Gleason grade.

### Paraffin signal removal and preprocessing

FFPE sections were mounted on CaF₂ substrates and covered with parafilm during handling to prevent dehydration. To correct for parafilm and paraffin contributions, we first acquired reference Raman spectra from (i) a standard parafilm sheet and (ii) a pure paraffin block under the same instrumental conditions as the tissue measurements. Parafilm‑related peaks were removed by software‑guided subtraction of the reference parafilm spectrum from the raw tissue spectra.

To further reduce paraffin contributions, we used SVD on the preprocessed spectra and identified the singular vector whose loading profile closely matched the reference paraffin spectrum (i.e., characteristic CH stretching and bending bands). This component was interpreted as the dominant paraffin‑like contribution. We then reconstructed the spectra after setting the corresponding singular value to zero, effectively removing that component from the data before downstream analysis. This procedure reduced wax‑related variance while preserving tissue‑specific biochemical features. All subsequent analyses (SVD, LDA, ROC, confusion matrices) were performed on these paraffin‑corrected spectra. Scattered Raman light from a laser beam focused on a tissue section provided detailed information about the molecular composition of the tissue at the microscopic level. Multiple spectra were obtained per tissue section to account for heterogeneity.

### Autofluorescence correction

All raw Raman spectra exhibited a broad fluorescence background arising from endogenous tissue fluorophores excited at 532 nm. To correct for this, we applied a two‑step autofluorescence suppression procedure. First, a low‑order polynomial baseline subtraction (LabSpec6) was performed to remove the smooth, slowly varying fluorescence background characteristic of flavins, porphyrins, lipofuscin, and other native fluorophores. Second, after baseline correction, singular value decomposition (SVD) was applied to the spectra, and singular vectors dominated by broad, featureless fluorescence profiles were identified and removed by zeroing their corresponding singular values during spectral reconstruction. This approach effectively reduced endogenous autofluorescence while preserving sharp Raman vibrational features used for downstream analysis.

### Raman data processing and analysis

HORIBA LabSpec6 software was used for the initial data processing: smoothing, baseline removal (polynomial), and normalization (unit vector), necessary to enable subsequent quantitative analysis [23–25]. SVD analysis of the spectra was done using Python code to obtain the critical spectral features differentiating between the tissue samples. All Raman spectra from each imaging dataset were aggregated to form an input matrix for the SVD algorithm. In particular, the Raman spectra acquired from individual points within each sample were organized to generate a matrix of size m × n, where m denotes the number of data points per spectrum, and n indicates the total number of spectra within a specific sample [37–40]. In our study, these dimensions were 584 × 80. Subsequently, we applied the SVD function in Python to decompose the input matrix into matrices U, Σ, and V^T^. The matrix V was utilized to create SVD scatter plots, whereas the individual SVD components were stored in the matrix U [41, 42]. Each scatter plot was based on the leading SVD components and contained two data sets, either a Gleason score (G7, G8, G9, G10) vs. control, or two Gleason scores (G7 vs. G8, G7 vs. G9, G7 vs. G10, G8 vs. G9, G8 vs. G10, G9 vs. G10), where each spectrum was represented as a single point. A separating line was constructed using Linear Discriminant Analysis (LDA), a supervised classification technique that identifies the linear combination of features which maximizes the separation between classes by maximizing the distance between their means while minimizing the variance within each class [43]. A corresponding confusion matrix, summarizing the classification performance for each sample, was also generated. Additionally, the SVD components employed in the scatter plots, each of which contained the spectral features responsible for distinguishing the datasets, were plotted.

### Exploratory discriminant analysis

Singular value decomposition (SVD) was applied to reduce the dimensionality of the dataset and to identify orthogonal components capturing the major sources of variance. The reduced feature space was then used as input for linear discriminant analysis (LDA), which identifies the linear combination of variables that maximally separates the predefined groups based on within‑ and between‑class covariance.

This analysis was conducted for exploratory purposes to assess class separability and underlying structure in the data. Because the goal was not to develop or validate a predictive model, no train–test split or cross‑validation procedure was performed. All discrimination metrics presented—including the confusion matrix and ROC curve reflect in‑sample performance of the LDA projection and are not intended to represent out‑of‑sample predictive accuracy.

### Statistical analysis

Statistical analysis was done using GraphPad Prism (v8; GraphPad Software, Boston, MA). The comparison between the specific Gleason scores (G7, G8, G9, G10) vs. control, and between Gleason scores (G7 vs. G8, G7 vs. G9, G7 vs. G10, G8 vs. G9, G8 vs. G10, G9 vs. G10) was done using a one-way ANOVA followed by the Tukey multiple comparison test. Statistical significance was set at *P* < 0.05.

Singular Value Decomposition (SVD) was used solely for visualization purposes, not for classification or model fitting. This approach inherently avoids overfitting, as SVD was not part of the predictive pipeline. We selected the first six components based on their cumulative explained variance, which captured the most informative structure in the data while minimizing noise. Linear Discriminant Analysis (LDA) was applied for classification. This helped assess generalizability and mitigate overfitting risks. The following flowchart represents our steps:

**Figure:**
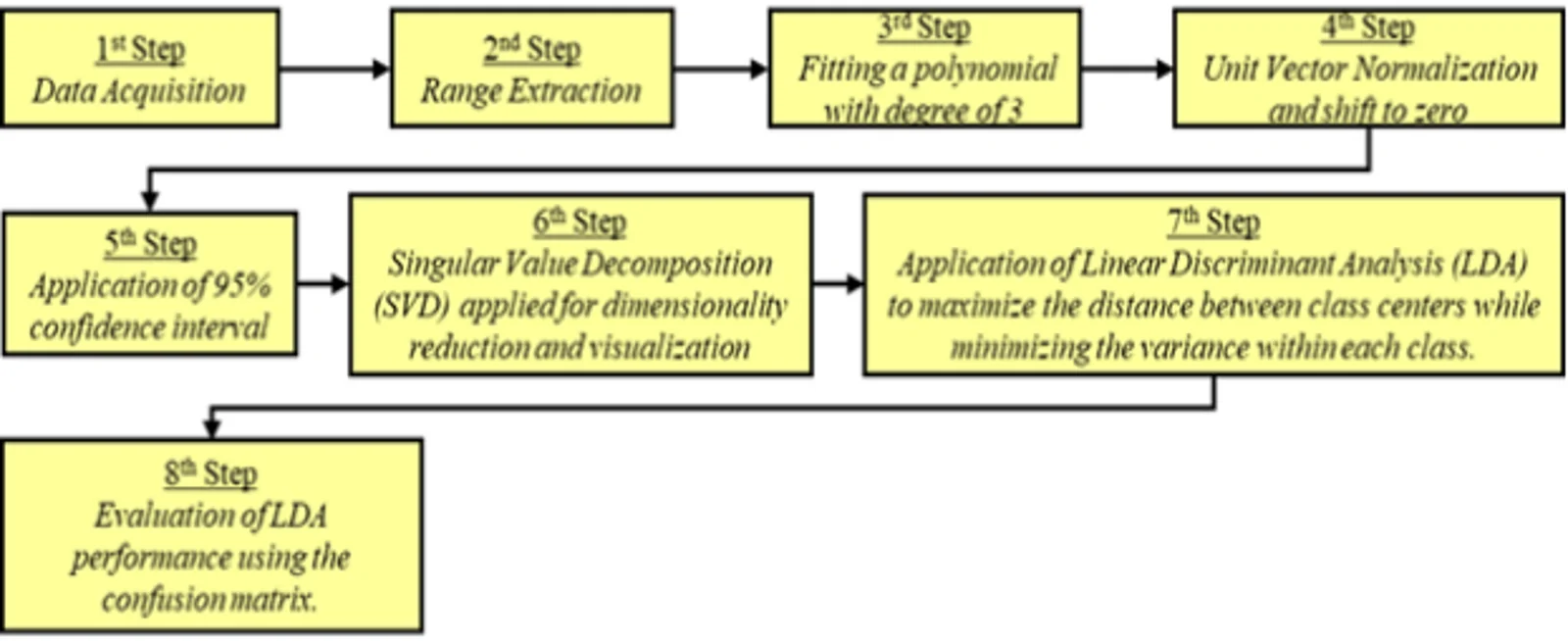

## Results

### Effect of residual paraffin signal

Although digital dewaxing substantially reduces paraffin contributions, residual overlap with wax bands cannot be completely excluded in FFPE tissues. Despite our efforts to subtract parafilm and suppress paraffin‑like components using reference spectra and SVD‑based removal, a low‑level residual wax contribution may still be present in the corrected spectra. However, several factors reduce the likelihood that this residual signal drives the observed class separation. First, all samples (controls and cancer of different Gleason grades) underwent identical FFPE processing, so any remaining paraffin contribution should be largely systematic across groups rather than class‑specific. Second, the SVD components most strongly associated with paraffin‑like features were explicitly identified and removed prior to classification, and the discriminative components used in LDA showed spectral features consistent with biological constituents (lipids, proteins, nucleic acids) rather than pure wax signatures. The SVD-based removal of the paraffin-associated component confirmed that the main Gleason-dependent trends in nucleic acid and protein bands remained unchanged, supporting the biological origin of the reported spectral differences.

### Raman spectral analysis

**Fig. 2:**
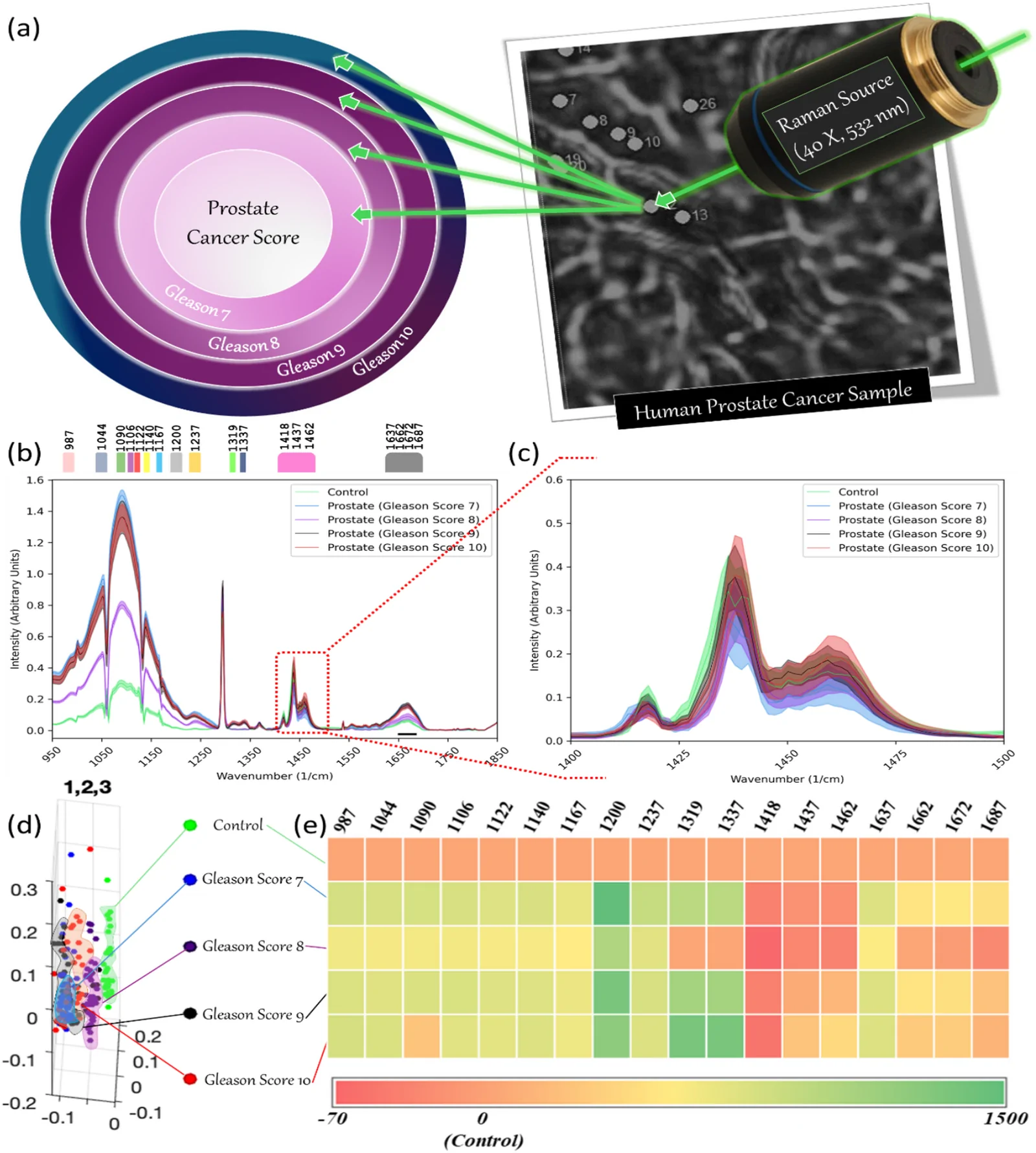
Representative Raman spectral analysis of CaP tissues with Gleason scores G7–G10 and normal controls. **a** Schematic overview of the Raman spectroscopy experimental setup. **b** Baseline-subtracted and normalized average Raman spectra for each Gleason score group and control, shown with 95% confidence intervals. **c** Inset highlights prominent spectral peaks within the 1400–1500 cm⁻¹ region. **d** Three-dimensional singular value decomposition (SVD) scatter plot illustrating spectral separation between control and Gleason score groups. **e** Heatmap depicting intensity variations of key Raman bands across Gleason scores

Figure 2 presents a comprehensive Raman spectral analysis of CaP tissues across Gleason scores 7 to 10, alongside normal prostate controls. The schematic in panel (a) outlines the experimental setup used for RS, highlighting the excitation source, sample positioning, and signal detection pathway. Panel (b) displays baseline-subtracted and normalized average Raman spectra for each Gleason group and control, with shaded regions indicating 95% confidence intervals. Notably, panel (c) zooms into the 1400–1500 cm⁻¹ region, revealing distinct spectral peaks associated with biochemical alterations in cancer progression. Panel (d) shows a three-dimensional SVD scatter plot, demonstrating clear spectral separation between normal and cancerous tissues, with increasing divergence correlating with higher Gleason scores. Finally, panel (e) presents a heatmap of Raman band intensities, illustrating progressive shifts in molecular signatures across the Gleason spectrum, underscoring the diagnostic potential of Raman spectroscopy in CaP grading.

Table 1 presents the key spectral changes observed in CaP tissue, emphasizing distinct Raman bands associated with Gleason scores G7 through G10 in comparison to non-cancerous control samples. These spectral shifts reflect molecular changes that correlate with disease severity and progression. Notably, specific Raman bands demonstrate consistent variation across increasing Gleason stages, suggesting their potential utility as biomarkers for tumor aggressiveness. The biological significance of each spectral feature, including its association with lipid metabolism, protein synthesis, and structural remodeling, is elaborated in the subsequent discussion.

In the RS analysis of CaP tissue, a prominent peak observed at 987 cm⁻¹ is indicative of molecular alterations associated with disease progression. This spectral feature is commonly linked to vibrational modes of cholesterol and cholesterol esters, which are known to accumulate in CaP cells, particularly in androgen receptor-negative subtypes [44]. The presence of this peak reflects a shift in lipid metabolism, a hallmark of cancerous transformation. Additionally, the 987 cm⁻¹ region may capture changes in protein structure and synthesis, as cancer cells often exhibit elevated protein turnover and remodeling.

**Table 1:** Comparative Raman band analysis in prostate cancer with respect to normal prostate control

Raman band (cm-1)	Intensity	Biological relevance
987	Decrease	Hydrogen atoms rocking vibrations in small peptides
1044 & 1090	Decrease	-O-P-O- (symmetric) of nucleic acids and C-C in phospholipids (with pH change)
1106	Decrease	Carbohydrates such as α-D-Glucose
1122, 1319 & 1584	Decrease	Cytochrome C characteristics band and Cytochrome C shoulder band; v(C-C) skeletal vibration of the acyl backbone in lipid (trans conformation)
1122 & 1140	Decrease	C-N stretch,* C-O/C-C stretch in proteins and carbohydrates*
1167	Decrease	C-H in-plane bending for aromatic amino acids in proteins
1205	Decrease	v(c-C_6_H_5_),phenylalanine, tryptophan in proteins; Adenosine, Thymine (ring breathing modes of the DNA/RNA bases) in nucleic acids; amide III (protein)
1237	Decrease	Amide III band in proteins; CH_2_ deformations in lipids
1337	Increase	*C-N signals from deoxynucleotides*
1418 & 1462	Increase	C-H asymmetrical bending mode of CH_2_/CH_3_ in lipids and proteins
1437, 1637 & 1687	Decrease	Characteristic band for L-Glutamate
1662	Decrease	v(C = C stretch) in lipids; amide I proteins; ring breathing v(pyrimidine) of nucleoside base
1437 & 1672	Increase	Specifically for nucleoside base (Thymine)

Significant spectral changes were observed in CaP tissue across Gleason scores 7 to 10, with notable shifts in Raman bands at 1044 cm⁻¹ and 1090 cm⁻¹. The 1044 cm⁻¹ band, attributed to phosphate vibrations from nucleic acids and phospholipids, showed increased intensity in higher Gleason grades, reflecting enhanced genomic activity and membrane remodeling associated with tumor progression [45–47]. Similarly, the 1090 cm⁻¹ band, linked to C–C and C–O stretching in lipids and carbohydrates, exhibited a progressive increase from Gleason 7 to 10, indicating metabolic reprogramming and lipid accumulation characteristic of aggressive CaP phenotypes [48, 49].

The observed increase in Raman intensity at 1106 cm⁻¹ across CaP tissues with Gleason scores 7 to 10 suggests a significant shift in cellular carbohydrate metabolism [50]. This band is closely associated with C–O and C–C stretching vibrations found in α-glucose and other carbohydrate structures. Its enhancement in higher-grade tumors may reflect elevated glucose uptake and utilization, consistent with the metabolic reprogramming characteristic of malignant transformation. The correlation between this spectral feature and α-glucose implies a potential link to the Warburg effect, wherein cancer cells preferentially metabolize glucose through glycolysis even under aerobic conditions. These findings support the role of the 1106 cm⁻¹ band as a biomarker for altered glucose dynamics and underscore its relevance in distinguishing aggressive CaP phenotypes [51–53].

Distinct spectral features at 1122 cm⁻¹, 1319 cm⁻¹, and 1584 cm⁻¹ were observed in CaP tissues and demonstrated increasing intensity with higher Gleason scores (G7–G10). The 1122 cm⁻¹ band is attributed to C–O stretching vibrations in carbohydrates and phospholipids, and its enhancement suggests altered membrane composition and glycosylation patterns associated with malignant transformation. The 1319 cm⁻¹ band corresponds to CH₂/CH₃ bending modes in lipids and proteins, indicating increased lipid turnover and structural remodeling in more aggressive tumors. The 1584 cm⁻¹ band is linked to C = C stretching in aromatic amino acids and nucleic acid bases, reflecting elevated protein synthesis and genomic activity in high-grade cancer [54, 55].

A notable increase in the Raman band at 1140 cm⁻¹ was observed in CaP tissues with escalating Gleason scores from 7 to 10. This band is primarily attributed to C–C and C–N stretching vibrations, commonly found in proteins, lipids, and amino acid side chains [53–56]. The progressive enhancement of this spectral feature suggests a shift in the biochemical composition of the tumor microenvironment, particularly reflecting increased protein synthesis and lipid remodeling associated with malignant transformation [56, 57].

A progressive increase in the Raman band at 1167 cm⁻¹ was observed in CaP tissues with advancing Gleason scores from 7 to 10. This band is primarily attributed to C–H in-plane bending and C–C stretching vibrations, commonly found in lipid chains and protein side groups [57–61]. The enhanced signal intensity at this frequency suggests elevated lipid turnover and protein conformational changes, both of which are characteristic of malignant transformation and tumor progression.

A progressive enhancement of the Raman band at 1205 cm⁻¹ was observed in CaP tissues with increasing Gleason scores from 7 to 10. This band is primarily attributed to C–C and C–N stretching vibrations, which are characteristic of protein backbones and lipid-associated structures [60, 61]. The elevated intensity at this frequency suggests increased protein synthesis, amino acid turnover, and membrane remodeling, all of which are hallmarks of malignant progression [62–65]. These biochemical changes support the metabolic demands of rapidly proliferating cancer cells and reflect the structural adaptations required for invasion and metastasis. In CaP, especially across Gleason scores 7 to 10, Amide III shifts may indicate structural remodeling of proteins- these shifts arise from N–H bending and C–N stretching in the protein backbone. In cancerous tissues, changes in this band may reflect protein conformational shifts and altered structural integrity [66].

Alternation in vibrational modes of the indole ring in tryptophan can signal oxidative stress, protein folding anomalies, or metabolic changes in tumor cells, and the symmetric ring breathing of the benzyl side chain in phenylalanine alters the reflects changes in protein expression, folding, and oxidative stress responses. Phenylalanine peak typically serves as a stable internal marker due to its sharp and consistent peak its increased intensity may reflect elevated protein synthesis or aromatic amino acid enrichment in cancerous tissues [67, 68].

A progressive increase in the Raman band at 1237 cm⁻¹ was observed in CaP tissues with advancing Gleason scores from 7 to 10. This band corresponds to the Amide III region, primarily arising from N–H bending and C–N stretching vibrations in the protein backbone. The enhanced signal at this frequency indicates alterations in protein secondary structure, such as shifts between α-helical and β-sheet conformations, which are commonly associated with malignant transformation [69–72]. These structural changes reflect the increased protein synthesis and turnover required to support rapid cellular proliferation and invasion in high-grade tumors.

The Raman band at 1337 cm⁻¹ is typically attributed to nucleic acid and protein vibrations, particularly related to adenine and CH deformation modes. In the context of CaP, higher Gleason scores often correlate with increased intensity or shifts in the 1337 cm⁻¹ band, due to changes in cellular composition. The 1337 cm⁻¹ band may reflect elevated nucleic acid content or altered protein structures, which are common in more aggressive tumors [73, 74].

Raman Band 1418 cm⁻¹ is attributed to CH₂ bending vibrations in lipids and proteins.

And in the context of CaP, a shift in this band may contribute to Changes in lipid composition and protein structure, which are common in malignant transformation. A decrease in lipid-associated bands like 1418 cm⁻¹ may indicate higher Gleason scores, reflecting more aggressive cancer. Lower intensity at this band may suggest loss of normal glandular architecture and increased cellular atypia. Raman Band 1462 cm⁻¹ is associated with CH₃ deformation modes, primarily from proteins and lipids, and this band has been observed to shift or change in intensity with increasing Gleason grade and potentially reflects altered protein synthesis or membrane composition in cancerous cells [75].

The 1437 cm⁻¹ Raman band is associated with CH₂ bending vibrations in lipids, and exhibited reduced intensity in higher-grade tumors, suggesting a depletion of lipid content consistent with malignant transformation. The 1637 cm⁻¹ band, attributed to amide I vibrations from protein secondary structures, showed spectral shifts and intensity changes indicative of altered protein conformation and synthesis in aggressive cancer phenotypes. Similarly, the 1687 cm⁻¹ band, also linked to amide I vibrations, demonstrated elevated signals in high Gleason score samples, reflecting increased cellular turnover and protein denaturation [74, 75]. The 1437 cm⁻¹ band is associated with CH₂ bending in lipids, which may be altered due to increased glutamine-driven lipid synthesis in cancer cells. Glutamine contributes to fatty acid biosynthesis, and changes in this band may reflect that metabolic shift. While the 1637 cm⁻¹ and 1687 cm⁻¹ bands correspond to amide I vibrations, linked to protein secondary structures. Elevated glutamine metabolism supports protein synthesis and turnover, especially in rapidly proliferating cancer cells; therefore, shifts or intensity changes in these bands may indicate increased protein content or altered folding, consistent with glutamine-driven growth.

The 1662 cm⁻¹ Raman band is attributed to amide I vibrations, particularly from C = O stretching in protein backbones. CaP cells often exhibit increased protein synthesis and turnover, leading to detectable changes in this band. The 1662 cm⁻¹ band intensity and position can vary with Gleason grade and, at lower Gleason scores (G7), tend to show more organized protein structures, with a stable amide I signal, while at higher Gleason scores (G8–G10) often exhibit increased or shifted 1662 cm⁻¹ signals, reflecting disordered protein structures and higher metabolic activity. In CaP, nucleoside metabolism is dysregulated, including increased synthesis and turnover of DNA and RNA, and while pyrimidine ring breathing itself is not captured in these bands, Raman shifts in the 1400–1700 cm⁻¹ range may reflect secondary biochemical effects of nucleic acid dysregulation such as altered protein expression, chromatin remodeling, and lipid biosynthesis [73, 74].

The 1437 cm⁻¹ band is attributed to CH₂ scissoring vibrations in lipid acyl chains and in CaP, where lipid metabolism is often disrupted, a decrease in intensity at 1437 cm⁻¹ has been observed in higher-grade tumors, reflecting reduced lipid content and altered membrane composition. Lower signal intensity at this band may indicate higher Gleason scores, as aggressive tumors tend to have diminished lipid signatures due to increased cellular proliferation and membrane remodeling. The 1672 cm⁻¹ Raman band falls within the amide I region, primarily associated with C = O stretching vibrations in protein backbones, and potential shifts or increased intensity at 1672 cm⁻¹ suggest changes in protein secondary structure, such as increased β-sheet content or protein denaturation, which are common in malignant tissues. Additionally, elevated or shifted signals at this band have been linked to higher Gleason grades, reflecting greater cellular atypia and metabolic activity. The 1672 cm⁻¹ band may reflect thymine’s C = O stretching, while 1437 cm⁻¹ is not directly linked to thymine but may indicate broader biochemical changes in CaP [73–75].

We should note that the artifacts around 1060 and 1140 cm-¹ are due to the digital paraffin removal procedure, and the broad feature observed near 1100 cm-¹ is likely influenced by residual paraffin contributions. This wavenumber region contains overlapping bands originating from both prostate tissue and paraffin, leading to partial spectral interference. The sharp peak near 1300 cm⁻¹ also corresponds to paraffin-related vibrational modes. The elevated baseline between 1050 and 1150 cm⁻¹ likely reflects a combination of tissue autofluorescence and residual paraffin contributions. Although baseline correction and digital dewaxing were applied, complete removal of wax-related background is challenging in FFPE sections and represents an inherent limitation of this sample type.

**Fig. 3:**
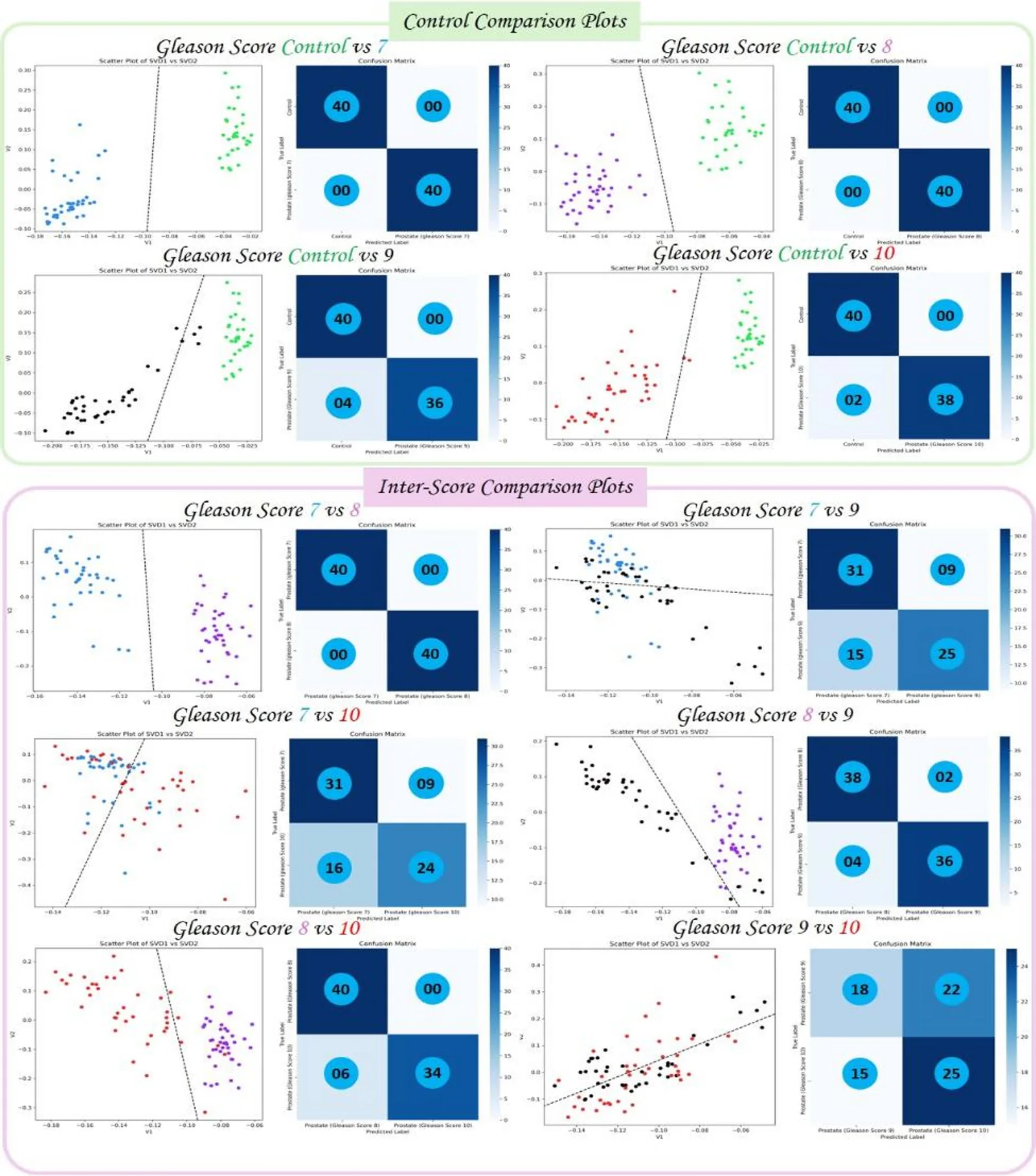
Singular value decomposition (SVD) plots demonstrate clear separation of Raman spectral clusters corresponding to different Gleason grades of CaP. The accompanying confusion matrices illustrate classification performance for each Gleason group, highlighting the accuracy of spectral differentiation across cancer grades. Confusion matrix showing in‑sample classification of all observations after SVD dimensionality reduction and LDA projection

Figure 3 illustrates the effectiveness of Raman spectral analysis in differentiating CaP tissues across Gleason grades by applying a Linear Discriminate Analysis (LDA), indicated by a dashed line, to the SVD scatter plots. The scatter plots reveal distinct clustering of spectral data corresponding to normal controls and Gleason scores 7 through 10, indicating clear biochemical separation among tissue types. Spectral clusters for higher Gleason grades (G9–G10) show greater divergence from normal tissue, reflecting increased molecular heterogeneity and malignancy. Accompanying confusion matrices quantify the LDA classification performance for each Gleason group, demonstrating high accuracy in spectral differentiation. The matrices highlight robust sensitivity and specificity, particularly for distinguishing high-grade CaP from lower-grade and normal tissues, underscoring the diagnostic potential of RS combined with multivariate analysis.

**Fig. 4:**
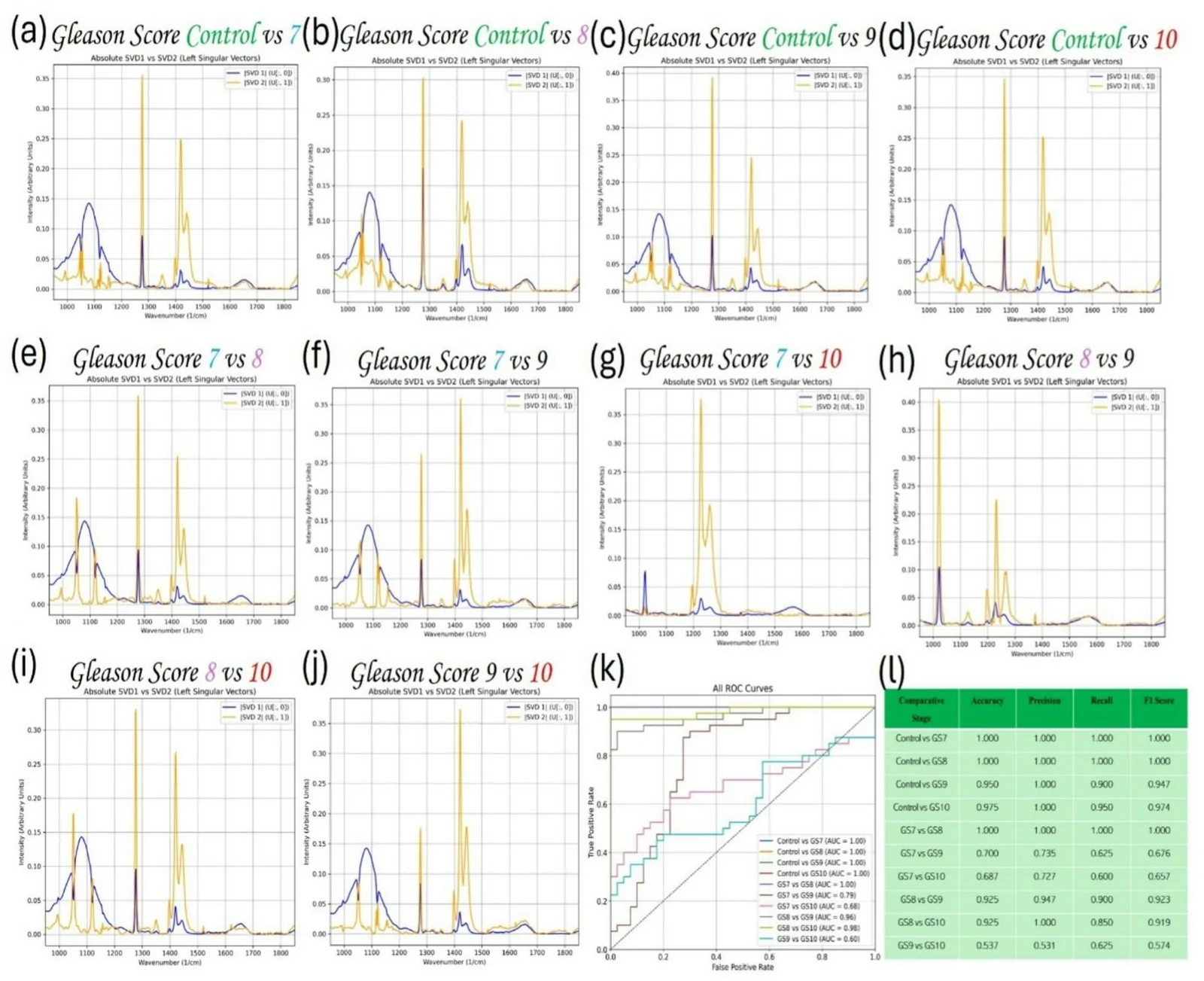
highlights the discriminative power of Raman spectroscopy in differentiating CaP tissues across Gleason scores and from normal controls. Singular vector component plots reveal distinct spectral variations, with clear differences between cancerous and non-cancerous tissues and among Gleason grades G7 to G10. These spectral distinctions reflect underlying biochemical changes associated with tumor progression and aggressiveness. Complementing these findings, receiver operating characteristic (ROC) curve analyses for each comparison dataset demonstrate high classification performance, with area under the curve (AUC) values indicating moderate sensitivity and specificity. Figure 4 shows that ROC curve analysis demonstrated excellent classification performance of the SVD-derived spectral features for distinguishing control samples from prostate cancer groups. All control versus Gleason score (GS) comparisons (Control vs. GS7, GS8, GS9, GS10) achieved perfect discrimination with an area under the curve (AUC) of 1.00, indicating complete separation between non-malignant and malignant spectra

Figure 4 Spectral differentiation and classification performance across Gleason score groups. (a–j) Pairwise comparisons of Raman spectral features between control and prostate cancer Gleason score groups (GS7, GS8, GS9, GS10) and between cancer grades. Each panel shows the absolute first (SVD1, blue) and second (SVD2, orange) left singular vectors derived from singular value decomposition (SVD) of the spectral datasets, highlighting dominant discriminatory features across the wavenumber range (∼1000–1800 cm⁻¹). Prominent peaks corresponding to biochemical constituents (e.g., proteins, lipids, nucleic acids) exhibit differential intensities across comparisons, reflecting progressive molecular alterations with increasing tumor grade. (k) Receiver operating characteristic (ROC) curves for all pairwise classifications, demonstrating the diagnostic performance of the SVD-based spectral features. Area under the curve (AUC) values indicate excellent discrimination for most comparisons, particularly between control and cancer groups. (l) Summary of classification metrics including accuracy, precision, recall, and F1-score for each comparison. Control versus cancer classifications achieve near-perfect performance (AUC ≈ 1.0), while discrimination between adjacent Gleason grades shows reduced but still moderate predictive capability, reflecting increasing biological similarity between higher-grade tumors.

Among cancer-only comparisons, classification performance varied with increasing similarity between adjacent Gleason grades. Comparisons between GS8 and higher grades showed strong discrimination, with AUC values of 0.96 (GS8 vs. GS9) and 0.98 (GS8 vs. GS10). Moderate classification performance was observed for GS7 vs. GS9 (AUC = 0.79) and GS7 vs. GS10 (AUC = 0.68). The lowest discrimination was noted for GS9 vs. GS10 (AUC = 0.60), indicating substantial spectral overlap between these advanced tumor grades. Overall, ROC curves consistently remained above the diagonal reference line, confirming predictive performance exceeding random classification across all comparisons. Together, these results underscore the feasibility of Raman spectroscopy as a non-invasive diagnostic tool for CaP grading and detection.

## Discussion

Our preprocessing pipeline substantially reduces wax‑related interference, however, a small residual paraffin contribution may persist in the corrected spectra. Several factors limit the likelihood that this residual signal influenced the observed class separation we see on Raman analysis. (1) all samples both controls and cancer tissues of varying Gleason grades, underwent identical FFPE processing and parafilm handling, meaning any remaining wax signal would be systematic across groups rather than class‑specific. (2) The two‑step correction procedure, which included subtraction of a reference parafilm spectrum followed by SVD‑based removal of the paraffin‑dominated singular vector, effectively suppressed the characteristic CH‑stretching and bending features associated with embedding media. The discriminative SVD components used for LDA contained sharp biochemical peaks consistent with proteins, lipids, and nucleic acids, rather than the broad or wax‑like features typical of paraffin. (3) LDA applied to the SVD‑reduced feature space demonstrated clear separation between the predefined groups. The resulting confusion matrix and ROC curve summarize the degree of discrimination achieved when the full dataset is projected onto the LDA axis. These metrics are presented in Fig. 4 solely illustrate in‑sample class separability. As the analysis was exploratory and not designed to generate a predictive model, these results should not be interpreted as independently validated classification performance. We explicitly affirm that no training/testing split or external validation was performed.

The RS analysis of CaP tissues across Gleason scores 7 to 10 revealed a series of biochemical alterations that reflect the progressive nature of malignancy. These spectral changes provide molecular insights into the metabolic reprogramming, structural remodeling, and genomic activity characteristic of aggressive CaP phenotypes.

A prominent peak at 987 cm⁻¹, indicative of lipid metabolism shifts and protein remodeling, underscores the early biochemical hallmarks of cancerous transformation. This band’s presence across Gleason grades suggests its potential utility as a marker for disease progression. Complementary changes in bands at 1044 cm⁻¹ and 1090 cm⁻¹ further support this, with increased intensity reflecting enhanced phosphate vibrations from nucleic acids and phospholipids, and C–C/C–O stretching in lipids and carbohydrates, respectively. These findings align with known patterns of membrane remodeling and genomic activation in high-grade tumors.

Carbohydrate metabolism also emerged as a key differentiator, with progressive increases in the 1106 cm⁻¹ band, which is associated with α-glucose structures, suggesting elevated glucose uptake and utilization. This is consistent with the Warburg effect and other metabolic shifts observed in malignancy. Additional carbohydrate- and lipid-associated bands at 1122 cm⁻¹, 1140 cm⁻¹, and 1167 cm⁻¹ demonstrated similar trends, reinforcing the role of glycosylation and membrane composition changes in tumor progression.

Protein-related bands showed a strong correlation with Gleason scores. The 1205 cm⁻¹ and 1237 cm⁻¹ bands, linked to C–C/C–N stretching and Amide III vibrations, respectively, increased with tumor grade, reflecting elevated protein synthesis and backbone remodeling. The 1337 cm⁻¹ band, associated with nucleic acid and protein vibrations, particularly adenine and CH deformation, also intensified in higher-grade tumors, suggesting increased genomic activity and altered protein structure.

Lipid-associated bands such as 1319 cm⁻¹, 1418 cm⁻¹, and 1437 cm⁻¹ exhibited reduced intensity in high Gleason scores, consistent with lipid depletion and membrane disruption. These changes may be driven by glutamine-dependent lipid synthesis pathways, which are upregulated in aggressive CaP. The 1462 cm⁻¹ band, linked to CH₃ deformation in proteins and lipids, showed shifts in intensity, further supporting altered membrane dynamics and protein turnover. Lipid-dominated bands at 1418, 1437, and 1462 cm⁻¹ showed reduced intensity in higher Gleason scores. While these modes partially overlap with paraffin vibrations in FFPE sections, their persistence and Gleason-dependent modulation after additional paraffin-component removal suggest a substantial contribution from tissue lipids and membrane-associated CH deformations.

The Amide I region bands, namely 1637 cm⁻¹, 1662 cm⁻¹, 1672 cm⁻¹, and 1687 cm⁻¹, demonstrated significant spectral shifts and intensity changes with increasing Gleason scores. These bands reflect changes in protein secondary structure, including increased β-sheet content and denaturation, indicative of elevated cellular turnover and metabolic stress. Notably, the 1672 cm⁻¹ band may also capture C = O stretching in thymine, suggesting a link to nucleic acid metabolism and DNA replication activity in high-grade tumors.

The RS findings presented in this study offer clinically significant insights into the biochemical landscape of CaP progression and its correlation with Gleason scores. The lipid-associated bands 987 cm⁻¹, 1090 cm⁻¹, 1437 cm⁻¹, and 1462 cm⁻¹ demonstrated reduced intensity in higher-grade tumors, reflecting disrupted lipid metabolism and membrane remodeling, which are hallmarks of aggressive cancer phenotypes. These changes suggest potential utility in identifying tumors with high proliferative capacity and altered energy storage pathways. Carbohydrate-related bands at 1106 cm⁻¹, 1122 cm⁻¹, and 1140 cm⁻¹ showed progressive enhancement with increasing Gleason scores, indicating elevated glucose uptake and glycosylation activity, which are biochemical features consistent with the metabolic reprogramming observed in malignancy. Protein structure and turnover were captured through amide-related bands at 1205 cm⁻¹, 1237 cm⁻¹, 1637 cm⁻¹, 1662 cm⁻¹, 1672 cm⁻¹, and 1687 cm⁻¹, which exhibited spectral shifts and intensity changes in high-grade tumors, reflecting increased protein synthesis, denaturation, and secondary structure alterations. These features are clinically relevant for assessing tumor aggressiveness and may inform therapeutic strategies targeting protein homeostasis. Nucleic acid-associated bands, including 1044 cm⁻¹, 1337 cm⁻¹, and 1584 cm⁻¹, showed increased intensity in higher Gleason scores, suggesting elevated genomic activity and DNA replication, which could aid in identifying tumors with high mutational burden or proliferative potential. Collectively, these spectral markers provide a molecular fingerprint of CaP progression and offer a non-invasive, objective complement to traditional histopathological Gleason scoring. Their integration into diagnostic workflows could enhance grading accuracy, reduce inter-observer variability, and support personalized treatment planning based on tumor biochemistry.

This study demonstrates the diagnostic utility of RS in grading CaP and distinguishing malignant from normal tissue. Gleason 7 cancers (3 + 4 and 4 + 3) were included to evaluate Raman based discrimination between subgroups that differ in the proportion of pattern 4. Figures 2 and 3, and 4 reveal progressive biochemical alterations across Gleason scores, captured through distinct spectral features. Figure 2 shows average Raman spectra and SVD-based separation, highlighting key peaks (1400–1500 cm⁻¹) linked to tumor-associated molecular changes.

Figure 3 presents SVD scatter plots with LDA clustering and confusion matrices, confirming accurate spectral classification, especially for high-grade CaP, and Fig. 4 further supports this conclusion with singular vector plots and ROC analyses, showing moderate sensitivity and specificity. The ROC analysis highlights the strong capability of SVD-based Raman spectral features to differentiate between benign and malignant prostate tissue with near-perfect accuracy. The AUC values of 1.00 for all control versus cancer comparisons suggest that malignant transformation is associated with pronounced biochemical alterations, likely reflecting changes in protein conformation, lipid composition, and nucleic acid content that are readily captured by the spectral decomposition approach. The near‑perfect ROC values in our study, compared with those reported by Aubertin et al. [26], likely reflect differences in tissue type, cohort composition, and analytical approach. Aubertin et al. [26]. examined fresh post‑prostatectomy specimens across Grade Groups 1–5, whereas our analysis used FFPE tissues and focused only on benign tissue versus higher‑grade cancers (Gleason ≥ 7), which show more pronounced biochemical divergence. In addition, our SVD‑based feature extraction isolates highly discriminative spectral variance, potentially enhancing classifier performance. Finally, our smaller, tightly controlled cohort likely exhibited less biological heterogeneity than the larger and more diverse cohort analyzed by Aubertin et al. These factors together explain the higher ROC values observed in our study.

In contrast, discrimination between cancer grades is more nuanced and reflects underlying tumor biology. The high performance observed for GS8 vs. GS9 and GS8 vs. GS10 suggests that intermediate-grade tumors retain distinct molecular signatures relative to more aggressive phenotypes. However, the reduced AUC values for GS7 vs. GS10 and particularly GS9 vs. GS10 indicate increasing molecular convergence at higher Gleason scores. This is consistent with the known histopathological continuum in advanced prostate cancer, where poorly differentiated tumors exhibit overlapping biochemical and structural features.

The relatively low AUC for GS9 vs. GS10 (0.60) suggests that distinguishing between these high-grade tumors using spectral features alone may be challenging, potentially requiring integration with additional biomarkers or multimodal approaches. Nevertheless, the ability to robustly separate control from cancer and to reasonably stratify intermediate grades supports the clinical potential of this approach for diagnostic and prognostic applications.

The SVD-based spectral decomposition highlights the progressive biochemical evolution of prostate cancer with increasing Gleason score. The pronounced separation observed in control versus cancer comparisons reflects fundamental molecular differences between benign and malignant tissues, including increased nucleic acid content, altered protein conformation, and lipid remodeling associated with tumorigenesis. These findings are consistent with known metabolic reprogramming and structural changes in cancer cells.

The increasing intensity and sharpening of spectral peaks with higher Gleason scores suggest a correlation between tumor aggressiveness and molecular heterogeneity. In particular, the prominent features captured by SVD2 likely represent localized biochemical alterations, such as increased nucleic acid turnover and protein structural changes, which become more pronounced in higher-grade tumors.

Among cancer-only comparisons, the ability to distinguish GS7 from GS8 indicates that early-stage tumor progression involves measurable biochemical shifts. However, the reduced separability observed in GS7 vs. GS9 and GS7 vs. GS10 comparisons suggests increasing overlap in molecular signatures as tumors evolve. This trend is most evident in GS9 vs. GS10, where near-complete overlap of SVD profiles indicates convergence toward a common high-grade biochemical phenotype. Such convergence is consistent with the dedifferentiated nature of advanced prostate cancers, where structural and metabolic distinctions between grades diminish.

Importantly, the complementary roles of SVD1 and SVD2 highlight the strength of this analytical approach. While SVD1 captures global spectral trends, SVD2 enhances sensitivity to subtle but diagnostically relevant variations. Together, these components provide a robust framework for identifying spectral biomarkers of disease progression.

These findings support the utility of SVD-based Raman spectral analysis for prostate cancer detection and grading, particularly for distinguishing benign from malignant tissue and identifying intermediate disease stages. However, the limited differentiation between high-grade tumors suggests that integration with additional molecular or imaging.

Collectively, these findings validate RS as a robust, non-invasive tool for CaP assessment and support the integration of RS with multivariate statistical techniques for non-invasive, label-free CaP detection and grading. The ability to differentiate between Gleason scores and identify high-grade malignancies offers promising clinical utility, particularly in guiding biopsy decisions, monitoring disease progression, and personalizing treatment strategies.

## Limitations of our study

Although our findings demonstrate clear Raman‑detectable biochemical differences across Gleason grades, we acknowledge that the study’s modest sample size represents an important limitation. Another major limitation of our study is the absence of Gleason score 6 (Grade Group 1) cancers, which prevents evaluation of Raman based discrimination between low grade and clinically significant disease this was due to the limited availability of Gleason score 6 cases in our archived tissue bank. Prostate tissue is inherently heterogeneous, with substantial patient‑to‑patient and region‑to‑region variability, and larger cohorts are typically required to establish population‑level Raman biomarkers with strong statistical generalizability. The present work was therefore designed as an exploratory, proof‑of‑concept investigation using pathologist‑annotated FFPE biopsy cores to evaluate whether Raman spectroscopy can detect consistent grade‑associated spectral trends despite this biological variability. By acquiring multiple spectra from spatially distinct, histologically validated regions within each core, we aimed to capture intratumoral heterogeneity while maintaining rigorous pathological correspondence.

Our use of SVD followed by LDA was appropriate for exploratory visualization and identification of dominant biochemical trends, however, future studies will need to incorporate additional multivariate and machine learning approaches such as PLS DA, random forests, or deep learning to improve predictive performance and generalizability. Additionally, larger patient cohorts are necessary to fully capture the biological variability inherent to prostate cancer, including patient to patient heterogeneity and regional differences within individual glands.

## Conclusion

CaP diagnosis and treatment strategies are based on the architecture of tissue structures, such as the Gleason score and the blood PSA levels. While biopsies and PSA levels are reliable technologies for oncologists due to their clinical utility, other alternatives that can capture the biochemical landscape of CaP progression are warranted. These involve identifying molecular changes, biomarkers, and proteomic shifts that signal disease advancement and recurrence. Our goal in this initial study was not to define definitive Raman biomarkers but to evaluate whether consistent biochemical differences across Gleason grades could be detected in FFPE tissue. The results of this exploratory study highlight the promising potential of Raman spectroscopy as a complementary tool for prostate cancer grading and risk stratification. By capturing subtle biochemical alterations associated with increasing Gleason grade, RS offers a molecular level perspective that is not accessible through conventional histopathology alone. Collectively, our findings highlight the utility of RS in capturing the biochemical landscape of CaP progression. The observed spectral trends across Gleason scores support the integration of Raman-based diagnostics into histopathological workflows, offering a non-invasive, molecularly informed approach to tumor grading. The clinical relevance of these RS findings lies in their potential to enhance CaP diagnosis, grading, and treatment planning through non-invasive biochemical profiling. Because RS can be applied to standard FFPE biopsy specimens without additional processing, it is readily compatible with existing clinical workflows and could be integrated into routine pathology pipelines or used as an adjunctive decision support tool.

## Future studies

Since RS offers a molecular level perspective that is not accessible through conventional histopathology alone, this capability may be particularly valuable in diagnostically challenging clinical scenarios such as distinguishing Gleason 3 + 4 from 4 + 3 or identifying early biochemical signatures of high-grade disease. Future studies incorporating expanded patient cohorts across the entire Gleason Score spectrum (G6-G10) and utilization of complementary classification methods will be used to validate and refine the Raman biomarkers identified here and to further assess their potential clinical utility. Although FFPE tissue provided a practical and clinically relevant platform, fixation and processing can introduce biochemical alterations; therefore, complementary studies using fresh or frozen tissue will be important to validate the robustness of the identified spectral markers. With further validation in larger, multi-institutional cohorts, RS based biomarkers have the potential to enhance diagnostic accuracy, reduce inter observer variability, and ultimately support more personalized treatment planning for patients with prostate cancer. Additionally, future studies will explore the integration of these spectral markers with machine learning models to enhance the accuracy and reproducibility of Gleason scoring.

## Data Availability

No datasets were generated or analysed during the current study.
